# Dual functionality of *O*-GlcNAc transferase is required for *Drosophila* development

**DOI:** 10.1098/rsob.150234

**Published:** 2015-12-16

**Authors:** Daniel Mariappa, Xiaowei Zheng, Marianne Schimpl, Olawale Raimi, Andrew T. Ferenbach, H.-Arno J. Müller, Daan M. F. van Aalten

**Affiliations:** 1 MRC Protein Phosphorylation and Ubiquitylation Unit, University of Dundee, Dundee, UK; 2 Division of Molecular Microbiology, University of Dundee, Dundee, UK; 3 Division of Cell and Developmental Biology, College of Life Sciences, University of Dundee, Dundee, UK

**Keywords:** *O*-GlcNAc, *O*-GlcNAc transferase, *Drosophila* development, *Hox*

## Abstract

Post-translational modification of intracellular proteins with *O*-linked *N*-acetylglucosamine (*O*-GlcNAc) catalysed by *O*-GlcNAc transferase (OGT) has been linked to regulation of diverse cellular functions. OGT possesses a C-terminal glycosyltransferase catalytic domain and N-terminal tetratricopeptide repeats that are implicated in protein–protein interactions. *Drosophila* OGT (*Dm*OGT) is encoded by *super sex combs* (*sxc*), mutants of which are pupal lethal. However, it is not clear if this phenotype is caused by reduction of *O*-GlcNAcylation. Here we use a genetic approach to demonstrate that post-pupal *Drosophila* development can proceed with negligible OGT catalysis, while early embryonic development is OGT activity-dependent. Structural and enzymatic comparison between human OGT (hOGT) and *Dm*OGT informed the rational design of *Dm*OGT point mutants with a range of reduced catalytic activities. Strikingly, a severely hypomorphic OGT mutant complements *sxc* pupal lethality. However, the hypomorphic OGT mutant-rescued progeny do not produce F2 adults, because a set of *Hox* genes is de-repressed in F2 embryos, resulting in homeotic phenotypes. Thus, OGT catalytic activity is required up to late pupal stages, while further development proceeds with severely reduced OGT activity.

## 1. Introduction

Post-translational modification of more than 1000 proteins with *O*-linked *N*-acetylglucosamine (*O*-GlcNAc) has been shown to affect a diverse array of cellular functions in metazoa, including protein stability, intracellular localization, protein–protein interaction, phosphorylation and ubiquitylation [1–3]. The enzyme that catalyses the addition of a single GlcNAc onto serine/threonine residues on intracellular proteins is *O*-GlcNAc transferase (OGT). OGT is highly conserved in metazoa, consisting of a C-terminal glycosyltransferase domain and N-terminal tetratricopeptide repeats (TPRs). Recent studies on the structure of the catalytic domain of human OGT (hOGT) have provided an insight into the mechanism of catalysis and protein substrate recognition [4–6]. TPRs are 34-amino acid helical protein–protein interaction motifs that are typically clustered in 3–16 repeats. TPR motif-containing proteins participate in a diverse array of functions, often as part of multi-protein complexes [7]. In hOGT, the TPRs adopt a right-hand super-helical conformation protruding away from the catalytic domain [5,8]. This TPR super-helix creates a large surface area that is thought to allow OGT to interact with a variety of protein substrates. Most OGT interactors require its TPRs for binding, for instance GRIF-1, OIP106 [9], Sin3A [10] and TET2 [11], while some others interact with the C-terminal domain as in the case of MAPK [12]. OGT isoforms possessing varying numbers of TPRs or recombinant OGT lacking the full complement of TPRs have distinct substrate preferences [13,14]. However, there is no evidence implicating the involvement of OGT in an interaction that does not ultimately invoke its catalytic activity. Nevertheless, it is plausible that proteins interacting only with the most N-terminal TPRs of OGT are non-substrate interactors.

Most animal genomes appear to contain a single *ogt* gene, except zebrafish, which has two [15]. In several animal models, it has been shown that *ogt* is essential for embryogenesis and early development. *ogt* null mice are embryonic lethal, while tissue-specific *ogt* knock out in T cells, fibroblasts and neurons results in severe phenotypic abnormalities and perinatal death [16,17]. *ogt* knockdown in zebrafish and *Xenopus laevis* embryos produces severe growth defects, shortened body axis and retarded nervous system development [18,19]. In *Drosophila*, *ogt* is known as *super sex combs* (*sxc*), mutants of which do not develop beyond the pharate adult stage. *sxc* belongs to the *Polycomb* group (*PcG*) genes that play key roles in developmental regulation, stem-cell maintenance and genomic imprinting [20–22]. In *Caenorhabditis elegans,* animals homozygous for a partial deletion of *ogt-1* are viable and fertile [23]. In *Arabidopsis*, unlike animal genomes, there are two genes, *SPINDLY* (*SPY*) and *SECRET AGENT* (*SEC*) coding for OGT with both overlapping and distinct functions. Double mutants of these *OGT* genes are embryonic lethal [24].

Many of the above studies used genetic approaches designed to generate organisms that are OGT protein null. Transheterozygotic *Drosophila* larvae with an *sxc^1^* allele have been reported to possess low levels of expression of a truncated form (lacking the C-terminal 165 amino acids) of OGT [20]. Another *sxc* allele, *sxc^6^*, is a protein null mutant. Larvae carrying two other *sxc* alleles, *sxc^4^* and *sxc^5^*, also express mutant OGTs at levels comparable with the wild-type [20,22]. These alleles carry a point mutation (N948I; *sxc^4^*) or a 19-amino acid C terminal deletion (Δ1031–1059; *sxc^5^*). The phenotypes of the *sxc^4^* and *sxc^5^* alleles are as severe as the null mutants, suggesting a role for the OGT catalytic domain in *Drosophila* development. While the catalytic activity of *Dm*OGT^Δ1031–1059^ has not been assessed, larval lysate from *sxc^4^/sxc^4^* possesses catalytic activity comparable with that of *sxc^2637^/sxc^2637^*, an OGT null mutant. Given the lack of mutant OGTs with impaired catalytic activity, phenotypes associated with partial loss of *O*-GlcNAc levels have not been explored.

Here, we use *Drosophila melanogaster* as a model organism to investigate the dependence of developmental pathways on *O*-GlcNAcylation. The structural and catalytic similarities between human OGT (hOGT) and *Dm*OGT (*Dm*OGT) were investigated using protein crystallography and enzymology. Structure-guided mutagenesis led to the identification of OGT mutants with varying levels of catalytic activity leading to generation of transgenic flies expressing catalytically impaired *Dm*OGTs. Strikingly, pupal lethality observed in *sxc* mutants was rescued by overexpressing either the OGT^WT^ or the catalytically inactive OGT^D955A^ mutant. However, the *sxc* F1 progeny rescued with OGT^D955A^ do not produce any F2 adults. F2 embryos from OGT^D955A^ rescue display derepression of a subset of *Hox* genes. These experiments reveal that OGT activity is required for development to pupal stages, while a severely hypomorphic form of OGT is sufficient to support later developmental processes dependent on zygotic *sxc* products.

## 2. Results

### 2.1. *Dm*OGT is structurally similar to hOGT

Sequence similarity between hOGT and *Dm*OGT is high, with 90% and approximately 80% sequence identity in the TPR region and the catalytic domain, respectively. The most variable domain is the ‘intervening domain’, a 100-amino acid insertion between the two Rossmann-folds that constitute the catalytic domain, the sequence identity being only 39%. No function has been attributed to the intervening domain despite structural information being available [5]. Additionally, the patch of basic residues (contained within hOGT 958–1001) proposed to interact with phosphatidylinositol-(3,4,5)-trisphosphate (PIP_3_) is not conserved in *Dm*OGT.

**Figure 1.**
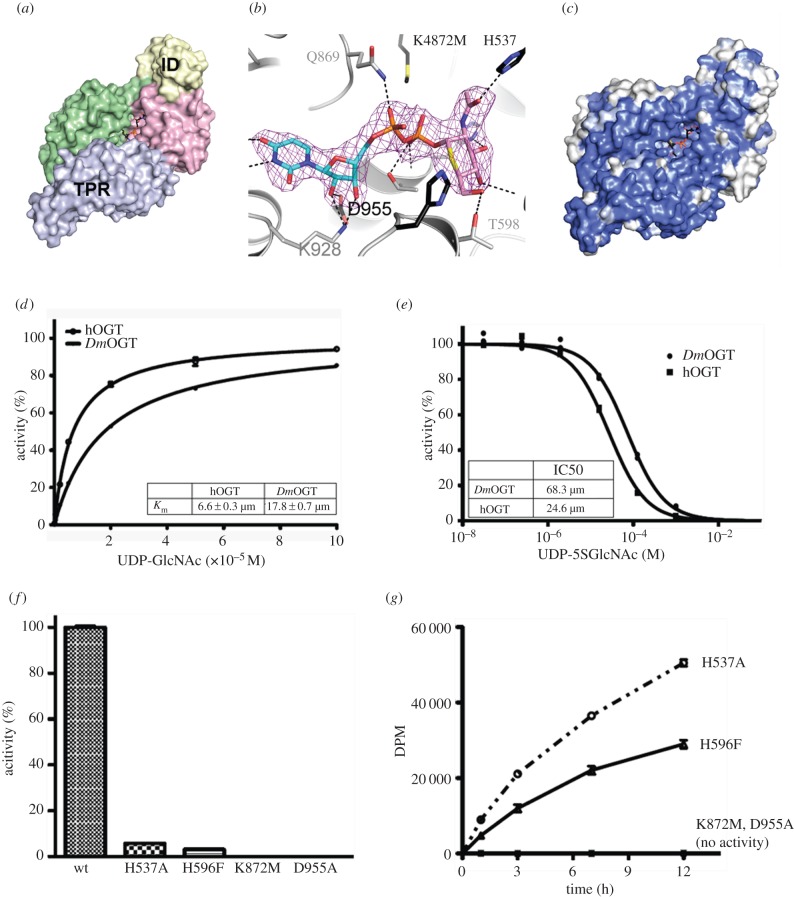
Structural and enzymatic characterization of *Dm*OGT. (*a*) *Dm*OGT adopts the canonical OGT fold with the intervening domain (ID, yellow surface) and the TPR repeats (grey surface) closely associated with the glycosyltransferase domain (green and pink surfaces). The donor substrate analogue UDP-5*S-*GlcNAc is shown as sticks with black carbons. (*b*) A close-up view of the catalytic site of *Dm*OGT with UDP-5*S-*GlcNAc shown as sticks with black carbon atoms. Unbiased F_o_-F_c_ electron density for the ligand is shown as pink mesh, contoured at 2.5 *σ*. The acceptor peptide TAB1tide from a superimposed hOGT structure (PDBID: 4AY6(6)) is shown as sticks with yellow carbon atoms. (*c*) The surface of *Dm*OGT coloured by sequence conservation with hOGT. Identical residues are shaded in blue, and non-conserved residues are shown in white. (*d*) Michaelis–Menten constants (*K*_m_) of UDP-GlcNAc for Δ1–352 *Dm*OGT and hOGT were determined in a radiometric *in vitro* assay on RBL2 peptide. Triplicate data points were fitted to the Michaelis–Menten equation. Error bars represent the standard error of the mean. (*e*) Half maximal inhibitory concentration (IC_50_) of UDP-5*S-*GlcNAc on Δ1–352 *Dm*OGT and hOGT were determined using the radiometric assay with UDP-GlcNAc concentration equal to the *K*_m_ for each enzyme. Duplicate data points were fitted to a three-parameter equation for dose-dependent inhibition. (*f*,*g*) The activities of recombinant Δ1–352 *Dm*OGT WT and the mutants were determined on RBL2 peptide *in vitro* using a radiometric assay.

To determine how differences in sequence influence the overall structure of *Dm*OGT and to provide a template for structure-guided mutagenesis, an N-terminally truncated construct starting at amino acid 353 in TPR 10 (Δ1–352) carrying a mutation (K872M) of a key catalytic lysine was expressed in *Escherichia coli* and crystallized in complex with the inhibitor/substrate analogue UDP-5*S-*GlcNAc. The structure was solved by molecular replacement and refined against 2.7 Å synchrotron diffraction data (table 1), yielding clear electron density for UDP-5*S-*GlcNAc (figure 1*a,b*). It appears that *Dm*OGT adopts the canonical OGT fold with the bilobal arrangement of two Rossmann-like domains characteristic of the GT-B superfamily of glycosyltransferases, as well as the additional TPR-like helices (535–566) in the N-terminal of the catalytic domain, which lead into the TPR domain (figure 1*a*). As a result, the TPRs are in close association with the glycosyltransferase domain and the catalytic site is aligned with the channel along the main axis of the TPR superhelix. Superposition of the catalytic domain of *Dm*OGT with the reported hOGT structure (PDBID 3PE4 (5)) highlights the structural similarities (RMSD = 1.35 Å for 660 Cαs). The catalytic site residues (H537, H596, Y871, K872, K928, H932, R935, H951, D955) that are found to interact with UDP-GlcNAc and the acceptor peptides in hOGT are conserved and similarly positioned in *Dm*OGT, suggesting a similar catalytic mechanism involving the same UDP-GlcNAc and acceptor peptide binding modes (figure 1*b*). Sequence conservation on the surface is most pronounced near the active site (figure 1*c*). The intervening domain, a 100-amino acid insertion between the two Rossmann-like folds of the catalytic domain, is the site of lowest sequence conservation and highest degree of flexibility in the structures of both hOGT and *Dm*OGT. Three short loops were disordered, but the overall domain architecture mirrors that of the hOGT intervening domain (figure 1*c*).

**Table 1.** Data collection and refinement statistics (values in brackets are those for the highest resolution bin).

	*Dm*OGT^K872M^
space group	*P*3_2_
cell dimensions	
* a* = *b*, *c* (Å)	160.95, 77.19
resolution (Å)	50.00—2.66 (2.75—2.66)
*R*_merge_	0.121 (0.916)
CC_1/2_	0.996 (0.627)
//*σ*/	8.8 (1.5)
completeness (%)	99.7 (99.1)
redundancy	5.1 (4.9)
no. reflections	327 309
*R*_work_/*R*_free_	0.225/0.264
no. atoms	
protein	16 023
UDP-5*S-*GlcNAc	114
water	99
average *B*-factors	
protein	50.8
UDP-5*S-*GlcNAc	39.5
water	37.5
RMSD from ideal geometry	
bond lengths (Å)	0.008
bond angles (°)	1.26

### 2.2. Rational design of catalytically impaired *Dm*OGT mutants

A recent study investigating the substrate sequence requirements of hOGT identified a peptide derived from human retinoblastoma-like protein 2 (RBL2; KENSPCVTPVSTA) as the best peptide substrate among the 720 Ser/Thr containing peptides [25]. RBL2 is a retinoblastoma family member—a family of proteins that are involved in a variety of cellular processes including the cell cycle, cell differentiation and apoptosis [26]. In order to explore the mechanistic similarities between the hOGT and *Dm*OGT, Michaelis–Menten kinetic parameters of the Δ1–352 *Dm*OGT were determined with the RBL2 peptide as an *in vitro* acceptor substrate in a radiometric assay. The Michaelis constant (*K*_m_) of UDP-GlcNAc for Δ1–352 *Dm*OGT is 17.8 ± 0.7 µM, similar to the *K*_m_ of the donor for Δ1–312 hOGT (6.6 ± 0.3 µM, figure 1*d*). A substrate-assisted glycosyltransfer mechanism has been proposed for hOGT, wherein a non-bridging oxygen of the *α*-phosphate of UDP-GlcNAc serves as the catalytic base [6]. This unique catalytic mechanism explains the specific inhibition of hOGT with the thiosugar derivative of the donor substrate, UDP-5*S-*GlcNAc [27]. Activity of the Δ1–352 *Dm*OGT is inhibited by UDP-5*S-*GlcNAc with an inhibition constant (*K*_i_) of 36.2 µM, comparable with the *K*_i_ of Δ1–313 hOGT at 13.6 µM (figure 1*e*). This observation further supports the hypothesis that *Dm*OGT adopts the same catalytic mechanism as hOGT, corroborating the structural data and Michaelis–Menten kinetics.

To identify OGT mutants with a range of catalytic activities, four *Dm*OGT point mutants were designed based on the structure of *Dm*OGT and previous enzymatic studies on hOGT [6,28,29]. The residue Asp925 in hOGT (*Dm*OGT^D955^) interacts with the ribose of the donor substrate (figure 1*b*) and an alanine mutant (D925A) has been shown to abolish hOGT enzymatic activity, primarily by disrupting UDP-GlcNAc binding [29]. Two other point mutants of hOGT, H558F (*Dm*OGT^H596F^) and K842M (*Dm*OGT^K872M^), have been reported to be enzymatically inactive while retaining the ability to bind the donor substrate UDP-GlcNAc with similar affinity as the wild-type enzyme [6]. In addition, H498A (*Dm*OGT^H537A^), a mutant of a residue that was previously thought to be important for catalysis, was chosen [5,6]. The Δ1–352 forms of *Dm*OGT^WT^ and the *Dm*OGT point mutants were expressed and purified. Enzymatic activities of these proteins were determined by a radiometric assay using the RBL2 peptide as acceptor substrate. Δ1–352 *Dm*OGT^K872M^ and Δ1–352 *Dm*OGT^D955A^ appear to have no catalytic activity even upon extending the assay to 12 h (figure 1*f*,*g*). However, Δ1–352 *Dm*OGT^H537A^ and Δ1–352 *Dm*OGT^H596F^ exhibited 5.6% and 3.0% activity, respectively, relative to Δ1–352 *Dm*OGT^WT^, and thus retained some degree of catalytic activity (figure 1*f*,*g*).

### 2.3. OGT catalytic activity is essential for early embryonic development in *Drosophila*

**Figure 2.**
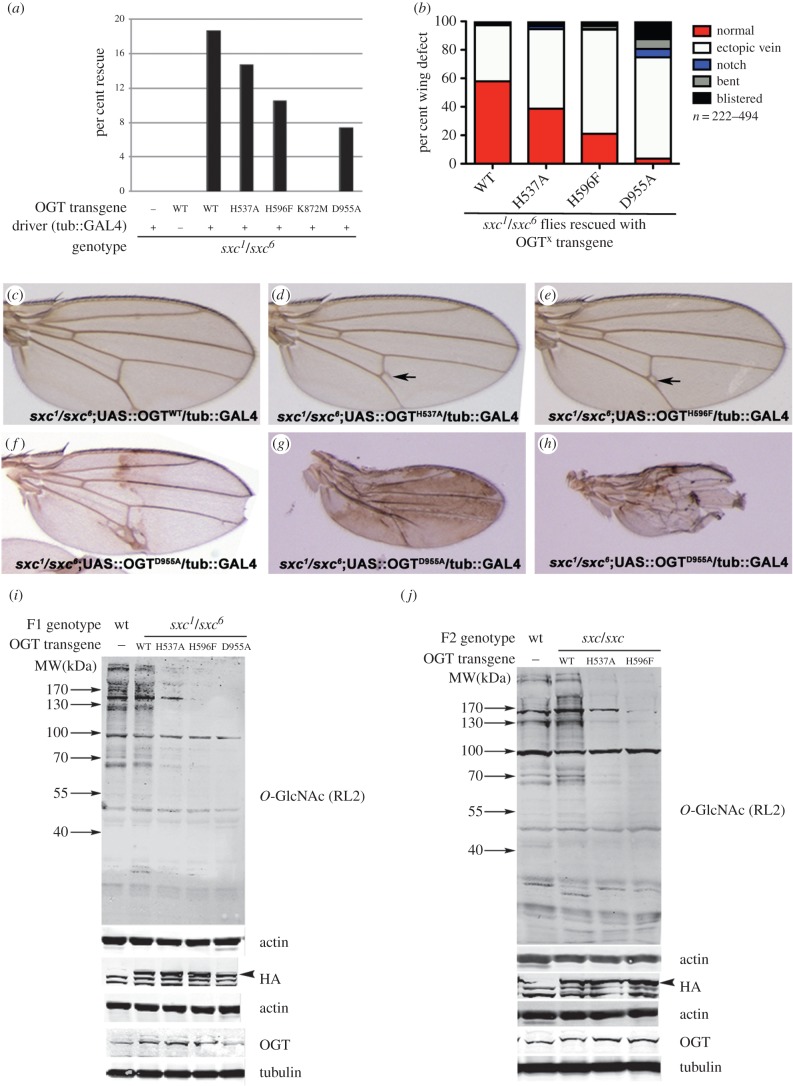
Catalytic activity of *Dm*OGT point mutants in flies. (*a*) Quantification of rescue to adulthood on driving *Dm*OGT transgenes in *sxc^1^/sxc^6^* mutants. (*b*) Quantification of wing phenotypes of *sxc^1^/sxc^6^* mutant flies rescued by driving the respective *Dm*OGT transgene. Both wings from the rescued flies were assessed for the following phenotypes: (*c*) normal wing, (*d*,*e*) ectopic vein, (*f*) notch, (*g*) bent or (*h*) blistered. (*i*) Total lysates from *w^1118^* (WT) or *sxc^1^/sxc^6^* transheterozygotes expressing the indicated UAS::OGT transgene under the control of tubulin::GAL4 were immunoblotted. The arrowhead points to the specific OGT-HA band. (*j*) Total lysates from *w^1118^* (WT) or *sxc/sxc* F2 flies derived from crosses between male and female F1 flies expressing the indicated UAS::OGT transgene under the control of tubulin::GAL4 were prepared and immunoblotted with the respective antibodies. As the F2 flies were derived from parents that are *sxc^1^/sxc^6^* and hence could have transheterozygotic (*sxc^1^/sxc^6^*) or homozygotic (*sxc^1^/sxc^1^ or sxc^6^/sxc^6^*) genotypes, they are indicated as *sxc/sxc*.

It has previously been demonstrated that transgenic expression of wild-type *Dm*OGT in *sxc* transheterozygotes rescues their lethality at the pharate adult stage [22]. Using this readout, the developmental requirements of catalytic versus non-catalytic functions of OGT were dissected (figure 2*a*). Rescue of the *sxc^1^/sxc^6^* pupal lethality was performed by ubiquitously driving the full-length *Dm*OGT^WT^ or one of the *Dm*OGT point mutants (H537A, H596F, K872M or D955A) with a tubulin::GAL4 driver. The details of all the genotypes obtained in these rescue experiments are outlined in table 2. The rescue measured with full-length *Dm*OGT^WT^ was in agreement with the previous report that also used a tubulin::GAL4 driver [22]. In the absence of this rescue, as is the case with control crosses lacking either the driver or the transgene, no adult *sxc^1^/sxc^6^* transheterozygotes were recovered. Of all the flies scored from the rescue cross, the fraction of *sxc^1^/sxc^6^* transheterozygote adults recovered on driving the *Dm*OGT^WT^ form of OGT ubiquitously was 18.6% (figure 2*a* and table 2). Given the crossing scheme, on complete rescue of the lethality phenotype, the rescued flies would constitute 20% of total progeny, which is in agreement with the level of *Dm*OGT^WT^ rescue observed. The fraction of rescued *sxc^1^/sxc^6^* transheterozygotes was 14.7%, 10.5%, 0% and 7.4% when the rescue was performed with *Dm*OGT^H537A^, *Dm*OGT^H596F^, *Dm*OGT^K872M^ and *Dm*OGT^D955A^ transgenes, respectively (figure 2*a*, table 2). Although the flies with the catalytically inactive transgene *Dm*OGT^D955A^ were rescued to adulthood, they had wing defects ranging from ectopic wing veins, small blisters, notches to severe blistering (figure 2*b*,*f*–*h*). While a high percentage (58%) of normal wings (figure 2*b*,*c*) were observed in flies rescued with the *Dm*OGT^WT^ transgene, 39% of wings had an ectopic vein (figure 2*b*). The proportion of flies with ectopic vein defects in *Dm*OGT^H537A^, *Dm*OGT^H596F^ and *Dm*OGT^D955A^ were 59%, 73% and 71%, respectively (figure 2*b*,*d*,*e*). More severe wing phenotypes were observed in *Dm*OGT^D955A^ rescued flies with a sizeable proportion (13%) of them being completely blistered (figure 2*b*,*f*–*h*). Quantification of the wing defects in the rescued adult flies revealed a correlation with the rescue efficiency and catalytic activity of the various constructs. *Dm*OGT constructs with higher rescue efficiency had less severe wing phenotypes (figure 2*a*,*b*). Crosses between F1 males and females of the same genotype (*sxc^1^/sxc^6;^tub::GAL4/UAS::OGT^X^*, where X = WT, H537A, H596F or D955A) obtained from the rescue crosses yielded F2 adults except in the case of *Dm*OGT^D955A^ (table 3)*.* To test whether the lack of F2 adults from the *Dm*OGT^D955A^ rescued *sxc^1^/sxc^6^* transheterozygous parents was a result of their infertility, rescued males/virgin females were crossed to wild-type virgins/males, respectively. While crosses using *Dm*OGT^D955A^ rescued *sxc^1^/sxc^6^* transheterozygote males produced adult progeny, the crosses with *Dm*OGT^D955A^ rescued *sxc^1^/sxc^6^* transheterozygote females only produced a few larvae that eventually died (table 3).

**Table 2.** Rescue of *sxc* lethality by OGT point mutants. Crosses were set up with flies of the indicated genotypes and transferred into fresh vials every 3–4 days. Adults emerging from the crosses were scored for the presence of second and third chromosome balancers/marker, CyO and MKRS or TM6. Flies that did not possess any of the balancers/markers (+;+) were the rescued *sxc^1^/sxc^6^* transheterozygotes. Control crosses with flies lacking either the driver (tubulin::GAL4) or any of the OGT transgenes do not yield any non-CyO adults. n.a., not applicable.

parental cross	total adults	CyO; TM6	CyO; MKRS	CyO; MKRS/TM6	CyO;+	+;+
*sxc^1^/CyO;MKRS/TM6* ♀ × *sxc^6^/CyO; tub::GAL4/TM6* ♂	512	162	177	173	n.a.	0
*sxc^1^/CyO;UAS::OGT^WT^* ♀ × *sxc^6^/CyO; MKRS/TM6* ♂	424	178	246	n.a.	n.a.	0
*sxc^1^/CyO;UAS::OGT^WT^* ♀ × *sxc^6^/CyO; tub::GAL4/TM6* ♂	376	226	n.a.	n.a.	103	70
*sxc^1^/CyO;UAS::OGT^H537A^* ♀ × *sxc^6^/CyO; tub::GAL4/TM6* ♂	565	348	n.a.	n.a.	134	83
*sxc^1^/CyO;UAS::OGT^H596F^* ♀ × *sxc^6^/CyO; tub::GAL4/TM6* ♂	462	317	n.a.	n.a.	99	46
*sxc^1^/CyO;UAS::OGT^K872M^* ♀ × *sxc^6^/CyO; tub::GAL4/TM6* ♂	590	459	n.a.	n.a.	131	0
*sxc^1^/CyO;UAS::OGT^N948I^* ♀ × *sxc^6^/CyO; tub::GAL4/TM6* ♂	441	401	n.a.	n.a.	40	0
*sxc^1^/CyO;UAS::OGT^D955A^* ♀ × *sxc^6^/CyO; tub::GAL4/TM6* ♂	495	323	n.a.	n.a.	136	36

**Table 3.** Maternal requirement of OGT catalytic activity. Crosses were set up using rescued F1 flies of the indicated genotypes and scored for the presence (+) or absence (−) of F2 adults or larvae. Wild-type males or females were also crossed with OGT^D955A^ rescued flies females or males, respectively to assess fertility of the F1 adults.

F1 cross	F2 larvae	adults
*sxc^1^/sxc^6^; UAS::OGT^WT^/tub::GAL4* ♀ × *sxc^1^/sxc^6^; UAS::OGT^WT^/tub::GAL4* ♂	+	+
*sxc^1^/sxc^6^; UAS::OGT^H537A^/tub::GAL4* ♀ × *sxc^1^/sxc^6^; UAS::OGT^H537A^ tub::GAL4* ♂	+	+
*sxc^1^/sxc^6^; UAS::OGT^H596F^/tub::GAL4* ♀ × *sxc^1^/sxc^6^; UAS::OGT^H596F^/tub::GAL4* ♂	+	+
*sxc^1^/sxc^6^; UAS::OGT^D955A^/tub::GAL4* ♀ × *sxc^1^/sxc^6^; UAS::OGT^D955A^/tub::GAL4* ♂	−	−
*sxc^1^/sxc^6^; UAS::OGT^D955A^/tub::GAL4* ♀ × *w1118* ♂	+	−
*w1118* ♀ × *sxc^1^/sxc^6^; UAS::OGT^D955A^/ tub::GAL4* ♂	+	+

To probe the level of catalytic activity in the *sxc* transheterozygotic animals rescued with the various transgenes, total protein *O*-GlcNAcylation was assessed by immunoblotting with anti-*O*-GlcNAc antibody (RL2) (figure 2*i*). Total *O*-GlcNAc levels were significantly reduced in flies rescued using the *Dm*OGT^H537A^ and *Dm*OGT^H596F^ mutants as opposed to complete restoration of *O*-GlcNAcylation in adults rescued with *Dm*OGT^WT^. Additionally, there was no detectable total protein *O*-GlcNAc in *sxc^1^/sxc^6^* animals rescued with *Dm*OGT^D955A^ (this assay could not be performed with flies rescued with *Dm*OGT^K872M^ because of the absence of viable flies). The level of OGT or OGT^mut^-HA expression was comparable across the lines (figure 2*i*). Total protein *O*-GlcNAc levels in F2 adults derived from *sxc* transheterozygotes rescued using *Dm*OGT^WT^, *Dm*OGT^H537A^ or *Dm*OGT^H596F^ were comparable with that of the F1 rescues (figure 2*j*). To determine the specificity of the signals obtained using the *O*-GlcNAc antibody (RL2), total WT fly lysates were incubated with *Cp*OGA, a potent enzyme that is known to remove *O*-GlcNAc from proteins [30,31]. *Cp*OGA treatment revealed that the prominent 100 kDa and lower molecular weight proteins are non-specific signals detected in the assay (electronic supplementary material, figure S1A). In addition, competition with 0.5 M GlcNAc during primary antibody incubation confirmed the specificity of RL2 reactivity (electronic supplementary material, figure S1B). These control experiments confirm that the *O*-GlcNAc-specific reactivity of RL2 is to proteins greater than 60 kDa.

**Figure 3.**
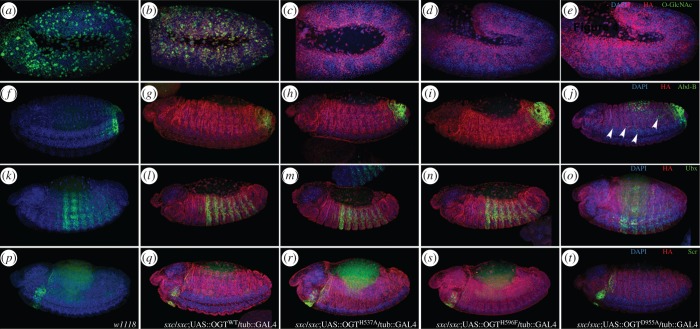
Repression of *Hox* genes in *Dm*OGT point mutant-rescued F2 embryos. Stage 9–11 (*a*–*e*) or stage 13–14 (*f*–*t*) F2 embryos were immunostained with *O*-GlcNAc (RL2; *a*–*e*), Abd-B (*f*–*j*), Ubx (*k*–*o*) or Scr (*p*–*t*) antibodies. F2 embryos from *w1118* (*a*,*f*,*k*,*p*) or crosses between F1 siblings with the following genotypes were collected, fixed and immunostained: *sxc^1^/sxc^6^;*UAS::OGT^WT^/tub::GAL4 (*b*,*g*,*l*,*q*), *sxc^1^/sxc^6^;*UAS::OGT^H537A^/tub::GAL4 (*c*,*h*,*m*,*r*), *sxc^1^/sxc^6^;*UAS::OGT^H596F^/tub::GAL4 (*d*,*i*,*n*,*s*) and *sxc^1^/sxc^6^*;UAS::OGT^D955A^/tub::GAL4 (*e*,*j*,*o*,*t*). White arrowheads indicate derepression of Abd-B in *sxc/sxc*;UAS::OGT^D955A^/tub::GAL4 embryos (*j*).

*sxc* has shown to be involved in *Polycomb* dependent derepression of *Hox* genes [32]. As observations can be confounded by the presence of maternal OGT products in the F1 embryos or larvae, phenotypes were assessed in F2 embryos. The F2 embryos are *sxc^1^* homozygotes, *sxc^6^* homozygotes or *sxc^1^/sxc^6^* transheterozygotes. Nevertheless, it is not possible to determine the precise *sxc* genotype on the second chromosome in these embryos. By immunostaining for the HA tag, the embryos expressing the respective OGT transgene can be identified and all experiments in rescued F2 embryos described further are in embryos expressing the respective *Dm*OGT transgene. *O*-GlcNAc levels were assessed in F2 embryos using the RL2 antibody. While *O*-GlcNAc levels in *Dm*OGT^WT^ rescued embryos were comparable with those in *w1118* embryos, faint immunostaining in cells overexpressing *Dm*OGT^H537A^ was observed (figure 3*a–c*). However, no *O*-GlcNAc staining could be observed in embryos rescued with either *Dm*OGT^H596F^ or *Dm*OGT^D955A^ (figure 3*d*,*e*). As *sxc* mutants display phenotypes similar to *PcG* genes that are involved in *Hox* gene repression, expression patterns of the *Hox* genes, *Sex combs reduced* (*Scr*), *Ultrabithorax* (*Ubx*) and *Abdominal-B* (*Abd-B*) were assessed by immunostaining. Expression patterns of Ubx (figure 3*k*–*o*) and Scr (figure 3*p*–*t*) remained unchanged in F2 embryos rescued with any of the *Dm*OGT transgenes and were comparable with the wild-type pattern. Interestingly, Abd-B was de-repressed anterior to its normal expression domain in most of the *Dm*OGT-D955A rescued F2 embryos (figure 3*j*). The Abd-B expression pattern was unaltered in all *Dm*OGT^WT^ and most of the *Dm*OGT^H537A^ and *Dm*OGT^H596F^ rescued embryos (figure 3*f*–*i*).

## 3. Discussion

The role of *sxc* as a *PcG* gene that functions in homeotic gene repression in *Drosophila* has been established [21]. The homeotic transformation and lethality phenotypes of *sxc* have been ascribed to the catalytic glycosyltransferase activity of OGT [20,22,32], but OGT is a large multi-domain protein known to participate in numerous protein–protein interactions [9–12]. We aimed to dissect the requirement of OGT catalytic activity during *Drosophila* development using a hypomorphic transgenic approach. The structure of *Dm*OGT was determined by X-ray crystallography, revealing that the overall fold and domain architecture of *Dm*OGT closely mirrors that of hOGT. The degree of flexibility of individual domains of OGT has been the subject of some speculation [5]. Central to this speculation is the assumption that OGT modifies intact, fully folded and predominantly *large* protein substrates. And yet, in the available structures of hOGT, the active site is not very accessible, being partly occluded by the TPR domain. This has led to the hypothesis of a ‘hinge-like movement’ between TPRs 12 and 13 that would expose the active site [5]. While such a substrate-induced conformational change cannot be excluded, the current structural evidence shows OGTs of three species (bacterial [29], human and *D. melanogaster*) adopting very similar conformations with limited access to the active site. In the absence of a complex between OGT and an entire protein, the question of substrate access remains unresolved.

The almost complete identity of sequence and structure in the active site of *Dm*OGT and hOGT suggested a similar catalytic mechanism and that any differences in substrate specificity are most probably attributable to regions of lower conservation beyond the active site. The largely unexplored intervening domain has the least structural similarity between hOGT and *Dm*OGT. We also noticed a higher degree of disorder in this part of the structure. If the intervening domain was involved in the recruitment of protein substrates, it would explain the flexibility we observed in the absence of a binding partner. A key observation from the sequence comparison of *Dm*OGT to hOGT is the non-conservation of the C-terminal array of lysines, which in hOGT was proposed to bind PIP_3_ and hence mediate translocation of hOGT to the plasma membrane upon insulin stimulation [33]. However, it has been recently reported that hOGT does not bind PIP_3_ [5]. The absence of a similar positively charged patch on the surface of *Dm*OGT further weakens the hypothesis that PIP_3_ binding in general could regulate the subcellular localization of OGT. Analysis of *Dm*OGT and hOGT structures guided the design of several OGT point mutants that were found to possess varying degrees of catalytic activities with *Dm*OGT^K872M^ and *Dm*OGT^D955A^ activities being undetectable. These mutations, by virtue of possessing almost no catalytic activity, provided tools to examine the role of OGT catalysis.

The TPR regions of OGT are better conserved than the rest of the protein. In keeping with this motif as a protein–protein interaction domain, there are several examples of OGT substrates interacting with the OGT TPR domain. Nevertheless, there are also examples of OGT catalytic domain-dependent binding of substrates. However, instances of OGT performing a scaffolding function completely independent of its catalytic activity are not known. Ectopic expression of the TPR domain was carried out in *Arabidopsis spindly* (*spy*) alleles, where lesions in TPRs are associated with gibberellin signalling defects [34]. However, it was reported that the TPR domain alone could not rescue the *spy* phenotype. When expressed in a wild-type background, the TPR domain mimics *spy* phenotypes, implicating that it disrupts interaction, oligomerization or localization of endogenous full-length SPINDLY [34]. Given that TPRs form the most prominent structural feature other than the catalytic core, OGT could also perform a non-enzymatic, scaffolding/adapter function as has been demonstrated for many other TPR proteins [35–38]. Nevertheless, one or more of the proteins assembling into such complexes could also be OGT substrates, thus complicating the dissection of these two functions. The only non-*O*-GlcNAc transfer role described for OGT is of aiding proteolysis of HCF1 and is dependent on the presence of the TPR domain as well as *O*-GlcNAcylation of HCF1 [39,40]. Notably, the key catalytic residue important for *O*-GlcNAc transfer, K842, is also essential for OGT proteolytic activity against HCF1.

Rescue of *sxc* pupal lethality is in agreement with a previous study [22] on rescuing with *Dm*OGT^WT^. While both *Dm*OGT^K872M^ and *Dm*OGT^D955A^ possess no detectable enzyme activity *in vitro*, the *sxc* phenotype could only be rescued in animals overexpressing *Dm*OGT^D955A^. Mutant forms of the equivalent residues in hOGT (K842M and D925A) have been shown to be inactive *in vitro* against peptide substrates [29,39]. Whereas hOGT^K842M^ can bind UDP-GlcNAc with similar affinity as the wild-type, hOGT^D925A^ was proposed to be deficient in donor substrate binding [29]. The difference in the rescue potential of *Dm*OGT^K872M^ and *Dm*OGT^D955A^ may be due to undetectably low activity of *Dm*OGT^D955A^. It therefore appears that adult *Drosophila* can survive with extremely low OGT catalytic activity. No F2 adults could be derived from *sxc* flies rescued with *Dm*OGT^D955A^ outlining the developmental requirement for catalytic activity in the absence of maternal products. However, minimal levels of OGT activity were sufficient to overcome this requirement, as evidenced by F2 progeny produced by *Dm*OGT^H596F^ *sxc* rescues.

Wing phenotypes in rescued F1 adults correspond to the level of OGT activity of the rescue construct, with reduced activity corresponding to more severe phenotypes. The most prevalent defect observed was for ectopic vein arising from the posterior cross-vein in rescues with almost all the constructs. Interestingly, similar ectopic vein phentotype, arising from the posterior cross-vein, is observed in *Drosophila HCF* (*dHCF*) mutants [41]. With dHCF being an OGT substrate, it is possible that sub-optimal *O*-GlcNAcylation of dHCF leads to the ectopic vein phenotype. Given that more than half of flies rescued even with *Dm*OGT^WT^ exhibit the ectopic vein phenotype, it implies that this phenotype is sensitive to precise levels of *O*-GlcNAcylation of an OGT substrate, possibly dHCF. Interestingly, other phenotypes observed in *dHCF* mutants [41] are not replicated in the rescued F1 adults.

Homeotic transformations in *sxc* mutants and the loss of PcG repression in larval imaginal discs have been demonstrated [20,21]. In *Drosophila*, components of polycomb repressive complex 1 (PRC1), a PcG complex, were found to be *O*-GlcNAcylated while the assembly of PRC2 was found to be upstream of *O*-GlcNAcylation in mouse ES cells [20,42]. More recently, however, it has been demonstrated in MCF-7 cells that the protein stability of EZH2, the histone methyl transferase component of PRC2, was dependent on its *O*-GlcNAcylation [43]. Nevertheless, recruitment of PRC proteins to DNA is not dependent on *O*-GlcNAcylation in *Drosophila* [20]. *O*-GlcNAcylation of YY1, a PcG protein, is essential for its interaction with retinoblastoma protein and hence transcriptional control by YY1 [44]. Polyhomeotic (Ph), a PcG protein, co-localizes with *O*-GlcNAcylation in polytene chromosomes and its recruitment to DNA at polycomb response elements is reduced in *sxc* mutants [20,22]. It has been demonstrated that when a Ser/Thr rich stretch in Ph is deleted, Abd-B is de-repressed, a phenotype also observed in *sxc^mat,zyg^* embryos [32]. Interestingly, *Ubx*, another *Hox* gene which was also reported to be de-repressed in *sxc^mat,zyg^* embryos [32], retains its normal domain of expression in the F2 embryos rescued with any of the *Dm*OGT transgenes in this study (figure 2*k*–*o*). This suggests that different mechanisms are possibly adopted by OGT to repress different *Hox* genes. Abd-B repression probably requires relatively higher OGT catalytic function as compared to repression of the other *Hox* genes.

In summary, we sought to determine the requirement for protein *O*-GlcNAcylation in *Drosophila* development. Rescue experiments in *sxc* mutants suggest the requirement of OGT catalytic activity up to early larval development. These experiments also demonstrate an extremely reduced requirement of OGT catalysis in pupal/adult stages. Association of the severity of wing phenotypes with reduced OGT activity implies a role for *O*-GlcNAcylated substrates in wing development. Embryos lacking maternal OGT catalytic activity, and consequently possessing significantly reduced *O*-GlcNAcylated substrates in the embryo, also display de-repression of only a subset of *Hox* genes. While these results underline the importance of OGT catalysis for *Drosophila* embryonic and larval development, it is intriguing that further development occurs almost independent of OGT catalytic activity. The possibility that non-catalytic OGT function(s) are essential in the adult remains unexplored.

## 4. Material and methods

### 4.1. Expression and purification of *Dm*OGT constructs

*Dm*OGT^Δ1–352^ K872M was expressed in ArcticExpress *E. coli* and purified as described for hOGT^Δ1–312^ [6].

### 4.2. Crystallization

Protein used in crystallization was stored in 25 mM Tris–HCl, pH 7.5, 150 mM NaCl and 2 mM DTT. Δ1–352 *Dm*OGT^K872M^ was crystallized in complex with UDP-5*S-*GlcNAc. Vapour diffusion crystallization experiments with sitting drops containing 0.2 µl protein (at 10 mg ml^−1^ in a buffer of 25 mM Tris–HCl pH 7.5, 150 mM NaCl, 2 mM DTT, 3 mM UDP-5*S-*GlcNAc) and 0.2 µl reservoir solution (condition 70 of the Molecular Dimensions Morpheus screen: 0.12 M mix of d-glucose, d-mannose, d-galactose, l-fucose, d-xylose, *N*-acetyl-dglucosamine, 30% PEG8000/ethylene glycerol and 0.1 M Tris–Bicine pH 8.5) gave needle-shaped crystals after 1–2 days at 21°C. The crystals were flash-frozen without further cryoprotection. Data were collected at the European Synchrotron Radiation Facility, Grenoble, France, on beamline ID29. Crystals belonged to space group *P*32 and contained 3 molecules per asymmetric unit. The structure was solved by molecular replacement with MOLREP [45] using the A chain of PDB ID 3PE4 and refined using iterative model building and refinement with COOT [46] and REFMAC5 [47] (table 1).

### 4.3. Enzyme kinetics on hOGT and *Dm*OGT

Steady–state kinetics was performed with the acceptor peptide KENSPCVTPVSTA using a radiometric assay. Assays were conducted in a final reaction volume of 20 µl containing 50 nM hOGT (Δ1–312) or 100 nM *Dm*OGT (Δ1–352), 0.25 mM RBL2 peptide and UDP-GlcNAc (100 µM, 50 µM, 20 µM, 5 µM, 2 µM, 0.5 µM and 0.05 µM) with final 0.3 Ci mmol^−1^ UDP-[^3^H]-GlcNAc as a radioactive tracer, in buffer containing 100 mM Tris–HCl, pH 7.5, 150 mM NaCl and 1 mM DTT. The reaction was stopped by addition of 200 µl of 0.75 M ice-cold phosphoric acid containing a final concentration of 0.5% trifluoroacetic acid (TFA) and 5% acetonitrile (ACN). A C18 column was activated by 200 µl 100% methanol and equilibrated twice with 200 µl of equilibration buffer containing 0.5% TFA and 5% ACN. Samples were passed through the C18 column by centrifugation at 500 r.p.m. for 30 s and columns were washed six times with 400 µl equilibration buffer. Peptide bound to the column was eluted by the addition of 100 µl 100% methanol followed by evaporation with SpeedVac. Radioactivity was detected by the addition of 4 ml scintillation fluid and signal was read by a scintillation counter. Triplicate data points were fitted to the Michaelis–Menten equation. Experiments to determine the half maximal inhibitory concentration (IC50) for UDP-5*S-*GlcNAc was performed as described above with UDP-GlcNAc concentrations fixed at the respective *K*_m_ for hOGT (5 µM) and *Dm*OGT (15 µM). Duplicate data points were fitted to a three-parameter equation for dose-dependent inhibition. The inhibition constants (*K*_i_) of UDP-5*S-*GlcNAc for both proteins were approximated by the Cheng–Prusoff equation.

### 4.4. *Drosophila* genetics, wing preparations and immunostaining

The following stocks from Bloomington Drosophila Stock Center were used: *w^1118^*, *sxc^1^/CyO, sxc^6^/CyO* and tub::GAL4/TM6. Transgenic flies were generated by Rainbow Transgenic Flies, Inc., California with the attP insertion site at 86F8. Rescue experiments were performed by crossing *sxc^1^/CyO* (*Kr::GFP*)*;*UAS::OGT^X^(X = either WT, H537A, H596F, K872M or D955A) and *sxc^1^/CyO* (*Kr::GFP*)*;*tub::GAL4/TM3 (twi::GAL4, UAS::2XEGFP), *Sb, Ser* flies. Rescued F1 flies of the genotype *sxc^1^/sxc^6^;*UAS::OGT^X^/tub::GAL4 (X = either WT, H537A, H596F or D955A) were assessed for the wing phenotypes or snap frozen for western blotting. Apart from wild-type wings, phenotypes included ectopic veins, notch, bent or blistered wings. Wings from rescued F1 *sxc^1^/sxc^6^;*UAS::OGT^X^/tub::GAL4 (X = either WT, H537A, H596F or D955A) adults were removed and transferred into isopropanol for 24 h. The wings were then mounted using DPX mounting medium (Fisher Scientific) after allowing for evaporation of isopropanol. Images were acquired using a dissection microscope after the medium was allowed to harden overnight. Fixing and immunostaining of embryos was performed as described previously [48]. The following antibodies were used: mouse anti-*O*-GlcNAc (1 : 250,RL2, Abcam), sheep anti-HA (1 : 500) and mouse antibodies from Developmental Studies Hybridoma Bank: anti-Scr (1 : 50), anti-Abd-B (1 : 50) and anti-Ubx (1 : 50) with the respective fluorescent secondary antibodies (Invitrogen). Microscopic images were obtained with a Zeiss 710 confocal microscope and processed using Volocity (Improvision) software.

### 4.5. Western blotting

For western blots, five anaesthetized adult flies were frozen on dry ice. The frozen flies were homogenized in 50 µl of lysis buffer (50 mM Tris–HCl, pH 8.0, 150 mM NaCl, 1% Triton-X-100, 1 µM GlcNAcstatin C, 5 mM sodium fluoride, 2 mM sodium orthovanadate, 1 mM benzamidine, 0.2 mM PMSF, 5 µM leupeptin and 1 mM DTT), following which an equal volume of three times SDS Laemmli buffer was added. Lysates were then heated for 5 min at 95°C, centrifuged at 16 000*g* for 10 min and supernatants were collected. Protein concentrations were estimated using the 660 nm protein assay (Thermo Scientific). Thirty micrograms of the crude lysate were subjected to SDS-PAGE and transferred onto nitrocellulose membrane before immunoblotting with RL2 (1 : 1000), rabbit anti-OGT (H-300, 1 : 1000, SantaCruz Biotech), mouse anti-HA (1 : 5000,12CA5) and/or anti-actin (1 : 5000, Sigma) and the respective infrared dye conjugated secondary antibodies (Li-Cor or Life Technologies, 1 : 10 000). To determine the specificity of the *O*-GlcNAc signal with the RL2 antibody, *w^1118^* adult fly lysates were treated with *Cp*OGA [30] for 30 min at 30°C before processing for western blotting. Alternatively, the RL2 antibody incubations were carried out in the presence of 0.5 M GlcNAc.

## Supplementary Material

Specificity of RL2 reactivity on adult Drosophila lysates

## Data accessibility

The *Dm*OGT structure has been submitted to the PDB database, with accession code 5A01.
