# Supplementary figures and images for: Dual functionality of O-GlcNAc transferase is required for Drosophila development
Source: Open Biol. 2015 Dec 16;5(12):150234. doi: 10.1098/rsob.150234 (PMC4703063; doi:10.1098/rsob.150234)

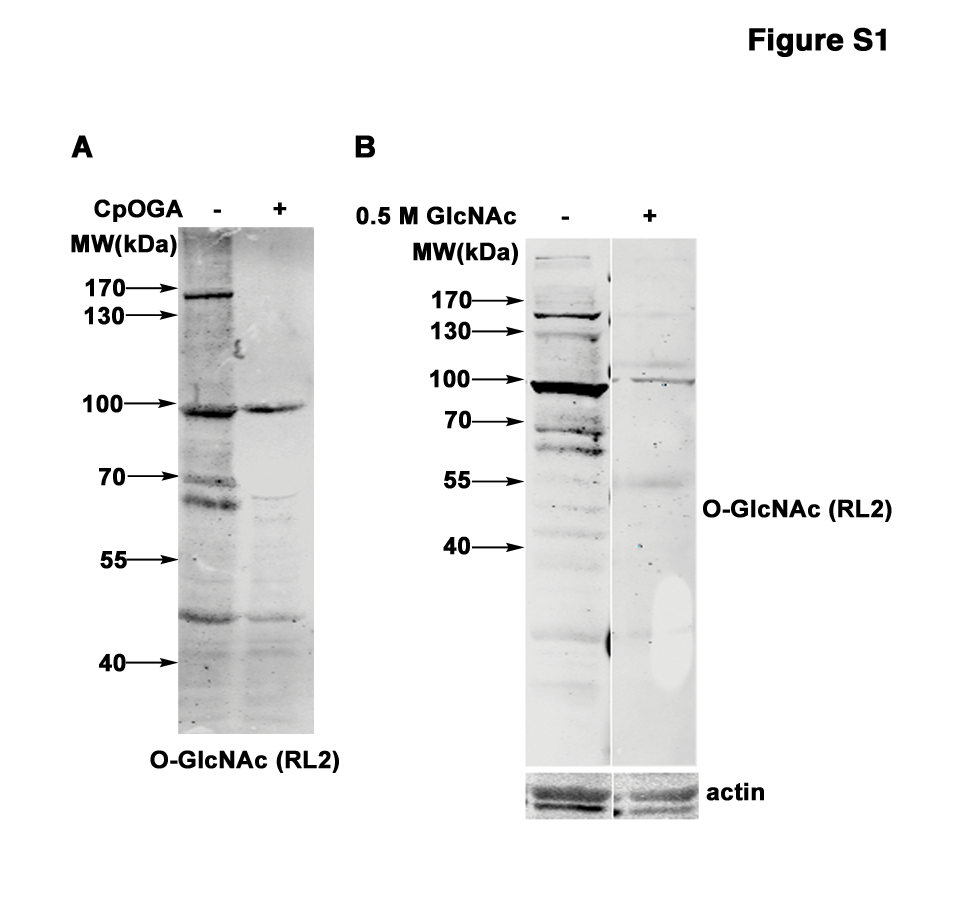

Supplement: Specificity of RL2 reactivity on adult Drosophila lysates [file rsob150234supp1.tif]
